# Bioceramics and Scaffolds: A Winning Combination for Tissue Engineering

**DOI:** 10.3389/fbioe.2015.00202

**Published:** 2015-12-17

**Authors:** Francesco Baino, Giorgia Novajra, Chiara Vitale-Brovarone

**Affiliations:** ^1^Department of Applied Science and Technology, Institute of Materials Physics and Engineering, Politecnico di Torino, Turin, Italy

**Keywords:** hydroxyapatite, calcium phosphate, bioglass, glass–ceramic, composite, bioactivity, porosity

## Abstract

In the last few decades, we have assisted to a general increase of elder population worldwide associated with age-related pathologies. Therefore, there is the need for new biomaterials that can substitute damaged tissues, stimulate the body’s own regenerative mechanisms, and promote tissue healing. Porous templates referred to as “scaffolds” are thought to be required for three-dimensional tissue growth. Bioceramics, a special set of fully, partially, or non-crystalline ceramics (e.g., calcium phosphates, bioactive glasses, and glass–ceramics) that are designed for the repair and reconstruction of diseased parts of the body, have high potential as scaffold materials. Traditionally, bioceramics have been used to fill and restore bone and dental defects (repair of hard tissues). More recently, this category of biomaterials has also revealed promising applications in the field of soft-tissue engineering. Starting with an overview of the fundamental requirements for tissue engineering scaffolds, this article provides a detailed picture on recent developments of porous bioceramics and composites, including a summary of common fabrication technologies and a critical analysis of structure–property and structure–function relationships. Areas of future research are highlighted at the end of this review, with special attention to the development of multifunctional scaffolds exploiting therapeutic ion/drug release and emerging applications beyond hard tissue repair.

## Introduction

The term “tissue engineering” was up to the mid 1980s loosely applied in the literature in cases of surgical manipulation of tissues and organs or in a broader sense when prosthetic devices or biomaterials were used. A clear definition was given by Langer and Vacanti (1993) as follows:

Tissue engineering is an interdisciplinary field that applies the principles of engineering and life science toward the development of biological substitutes that restore, maintain, or improve the tissue function.

Without a doubt, tissue engineering aims to provide a permanent solution to the replacement of tissues that are either defective or have been lost due to different pathological conditions, and it has emerged as a promising alternative to tissue or organ transplantation. This approach uses interdisciplinary tools to produce devices that have the potential to integrate and regenerate a specific functional tissue upon implantation. A key component of this strategy is a synthetic framework referred to as scaffold, which serves as a guiding two- or three-dimensional (2- or 3-D) structure for both hard- and soft-tissue development both *in vitro* and *in vivo*. Due to its open system of interconnected pores, the scaffold provides a mechanically stable environment that can host the required cells and biological components (seeded in the laboratory prior to implantation), allow cell migration, adhesion and growth, and support the organization of the growing tissue when implanted *in vivo* (Nerem, 1991). This is further enhanced by the use of “signaling,” which is another building block of tissue engineering. Signaling involves biochemical and biomechanical signals (delivered by the scaffold), which activate *in vivo* mechanisms of tissue regeneration, coaxing the cells into creating viable tissues and, thus, determining whether the scaffold turns into integrated tissue (Rutenberg et al., 2004; Johnson et al., 2007).

Many different materials have been investigated and engineered (natural and synthetic, bioresorbable, and permanent) to construct scaffolds. Among these, bioceramics have been extensively considered since these materials generally show better tissue responses compared to polymers and metals (Hench, 1998). Some bioceramics, such as hydroxyapatite (HA) and alumina, are intended to be permanent devices, thus they do not release their components into the human body and are expected to generate no foreign body reactions. On the other hand, if designed as resorbable biomaterials (e.g., most bioactive glasses) with various resorption kinetics (from days to months), their ion dissolution products (typically Ca, Si, Na, and phosphate ions) can be usually processed via normal metabolism (Habibovic and Barralet, 2011) or even exploited to exert a desired therapeutic effect, such as promotion of angiogenesis and antibacterial properties (Gerhardt et al., 2011; Hoppe et al., 2011; Mourino et al., 2012; Vargas et al., 2013).

Given the inorganic nature and mechanical rigidity of bioceramics, their traditional fields of application have been related to hard tissue repair, such as bone and teeth. However, several studies have also demonstrated the potential of bioceramics as an innovative route to regenerate various types of damaged soft tissues (Baino et al., 2016b; Miguez-Pacheco et al., 2015a).

This article will look at bioceramic materials used as scaffolds for hard- and soft-tissue engineering. First, basic scaffold requirements are examined and an overview of bioceramics used to produce a variety of scaffolds is given. Then, the main fabrication technologies used for making scaffolds are presented discussing both advantages and limitations. Further directions for the research are finally discussed, highlighting the promise of multifunctional engineered systems that combine the “conventional” proprieties of bioceramics and new, smart added values for improved therapeutic action (e.g., ion release and drug delivery).

## Scaffold Requirements and Critical Issues

Tissue engineering scaffolds have been widely studied with the hope of designing implantable biomaterials that can produce the most appropriate host response in which the clinical situation demands while supporting the growth and regeneration of complex 3-D tissues. There are several widely accepted requirements that should characterize an ideal scaffold (Hutmacher, 2000; Jones et al., 2007; Gerhardt and Boccaccini, 2010; Baino and Vitale-Brovarone, 2011), as summarized in Table 1.

**Table 1:** **Design criteria for tissue engineering scaffolds**.

Requirements	Description
(i) Geometry	It must initially fill complex 3-D defects, subsequently guiding the tissue to match the original 3-D anatomy
(ii) Bioactivity	Stimulation of rapid tissue attachment to the implant surface (without formation of scar/fibrous tissue) and creation of a stable long-term bonding that prevents micromotion at the interface and the onset of an inflammatory response
(iii) Biocompatibility	Ability to support normal cellular activity including molecular signaling systems without any local and/or systemic toxic effects to the host tissue
(iv) Chemical and biological stability/biodegradability	Depending on the specific application; if the scaffold must remain *in situ* indefinitely, materials with high stability must be selected; conversely, if it is intended to be a temporary device, the scaffold must degrade gradually over a predetermined period of time and be replaced by the natural host tissue
(v) Porous structure	The scaffold must possess an interconnected porous structure with a large surface-to-volume ratio and pore size of at least 100 μm in diameter (ideal for bone repair) to allow cell penetration, tissue in-growth, facilitate vascularization of the construct, and nutrient transport
(vi) Mechanical competence/compliance	The mechanical performance of the scaffold, which is determined by both the properties of the biomaterial and the porous structure, must be sufficient to withstand implantation handling and support the loads and stresses that the new tissue will ultimately bear. Adequate elastic compliance (low stiffness) with soft tissue is required for non-osseous applications
(vii) Biological properties	Special properties, such as the promotion of angiogenesis, stimulation of cell differentiation, and antibacterial effect, can be achieved by the release of appropriate ions from the scaffold material. These added values are typically imparted to bioactive glass scaffolds by carefully designing the glass composition
(viii) Fabrication	The scaffold should be easily tailored in size and shape to the diseased or injured area that the new tissue will replace
(ix) Commercialization potential	The scaffold should be produced with an automated technique in a reproducible manner; it should be fabricated and sterilized according to international standards for commercial production and clinical use

A major difficulty in the design of scaffolds is to simultaneously tailor these requirements due to their competing nature in fulfilling host tissue demands, namely, if a specific requisite is accomplished, another one might in turn be negatively affected.

A crucial aspect for the successful outcome of scaffolds for load-bearing applications (e.g., bone tissue repair) is the need to balance the porosity of a scaffold with its mechanical proprieties (Vitale-Brovarone et al., 2009). As described in Table 1, a highly interconnected porous structure (typically a pore content above 50 vol.%) is essential to enable full integration of the scaffold once it is implanted. However, porosity affects the mechanical competence of the component, as strength and stiffness progressively diminish when the volume fraction of porosity is increased (Gibson, 1989).

If the scaffold is intended to be bioresorbable, the achievement of mechanical competence becomes a further hurdle since degradable materials tend to be mechanically more and more fragile over time. Additional complications in the development of bioresorbable scaffolds are (i) the maintenance of strength and stability of the interface during the degradation period and replacement by the natural host tissue and (ii) matching the rate of resorption with that of the expected specific tissue regeneration. It is also important that the breakdown products of the biomaterial can be readily metabolized without causing any local or systemic adverse reaction (Hoppe et al., 2011).

Another aspect that is worth mentioning concerns the limitation of elastic modulus mismatch, which is crucial for the long-term success of implant bonding to both hard and soft tissues. Several studies demonstrated that the discontinuous change in elastic properties at the tissue–implant interface results in large stress gradients to the host tissue ultimately leading to failure of the implanted material (Hench and Greenspan, 2013).

Optimization of all the physicochemical parameters summarized in Table 1 is an extremely difficult task due to their complex and still partially unexplained interlocking. The rate and quality of tissue integration have been related to a dependence on scaffold pore size, porosity volume fraction, and pore interconnection (Karageorgiu and Kaplan, 2005). Moreover, the role of strut microstructure and pore geometry has to be considered with respect to their influence on entrapment and recruitment of growth factors in addition to their influence on scaffold mechanics. Deconvoluting the relative effects of these parameters is complicated by the bioactivity of many bioceramics, which is mediated through two principal mechanisms: (i) directly through dissolution and release of ionic products *in vitro* and *in vivo*, elevating local concentrations of soluble species that interact directly with local cells or influence cell behavior by their effect on local pH and (ii) indirectly through the influence that surface chemistry will have on protein adsorption, growth factor entrapment, and subsequent cell attachment and function. A valuable picture on these important issues in view of optimizing scaffold design and fabrication has been recently given by Hing (2005).

A highly challenging field of research concerns the strategies for imparting special “biological” properties to tissue engineering scaffolds, with particular reference to the use of bioactive glasses. It has been demonstrated that key mechanisms leading to enhanced new bone growth are related to the controlled release of ionic dissolution products (e.g., soluble silica and calcium ions) from the degrading bioactive glass (Hench, 2009). Specifically, a series of studies have shown that bioactive silicate glasses and their ionic dissolution products enhance osteogenesis by regulating osteoblast proliferation, differentiation, and gene expression (Xynos et al., 2000, 2001; Jell and Stevens, 2006; Jell et al., 2008). Sun et al. (2007) showed that 45S5 Bioglass^®^ promotes human osteoblast proliferation: in the presence of critical concentrations of Si and Ca ions, within 48 h osteoblasts that are capable of differentiating into a mature osteocyte phenotype begin to proliferate and regenerate new bone and, at the same time, osteoblasts that are not in the correct phase of the cell cycle and unable to proceed toward differentiation are switched into apoptosis by the ionic dissolution products.

The relative contribution of specific ion dissolution products from bioactive glasses or Si-substituted calcium phosphates to osteogenesis have been controversially debated in the literature (Bohner, 2009; Hoppe et al., 2011). It has been hypothesized that the high Si concentration from bioactive glass could be a major factor in stimulating osteoblasts to grow quickly, which might be effective for melt-derived bioactive glasses (Xynos et al., 2001; Sun et al., 2007). However, Bielby et al. (2004) found no significant differences in the proliferation of human primary osteoblasts grown in conditioned cell culture media containing similar Ca, P, and Na ions but different Si ion concentrations released from a sol–gel bioactive glass. Therefore, further studies are required to gain quantitative knowledge and to confirm the mechanisms by which ion dissolution products from bioactive glass may affect gene expression in bone cells.

Recent findings also indicate that controlled release of low concentrations of ionic dissolution products from bioactive glasses can induce angiogenesis that plays a key role in the regeneration process of both hard and soft tissue (Gerhardt et al., 2011; Vargas et al., 2013). The role of angiogenic and osteogenic factors in the adaptive response and interaction of osteoblasts and endothelial cells during the processes of bone development and bone repair has been reviewed in detail by Kanczler and Oreffo (2008).

Early studies suggesting that the ability of bioactive glasses to induce differentiation of non-osseous cells (e.g., muscle precursor cells exposed to phosphate glasses) have been recently reported (Ahmed et al., 2004).

## Bioceramics: A Short Overview

**Figure 1:**
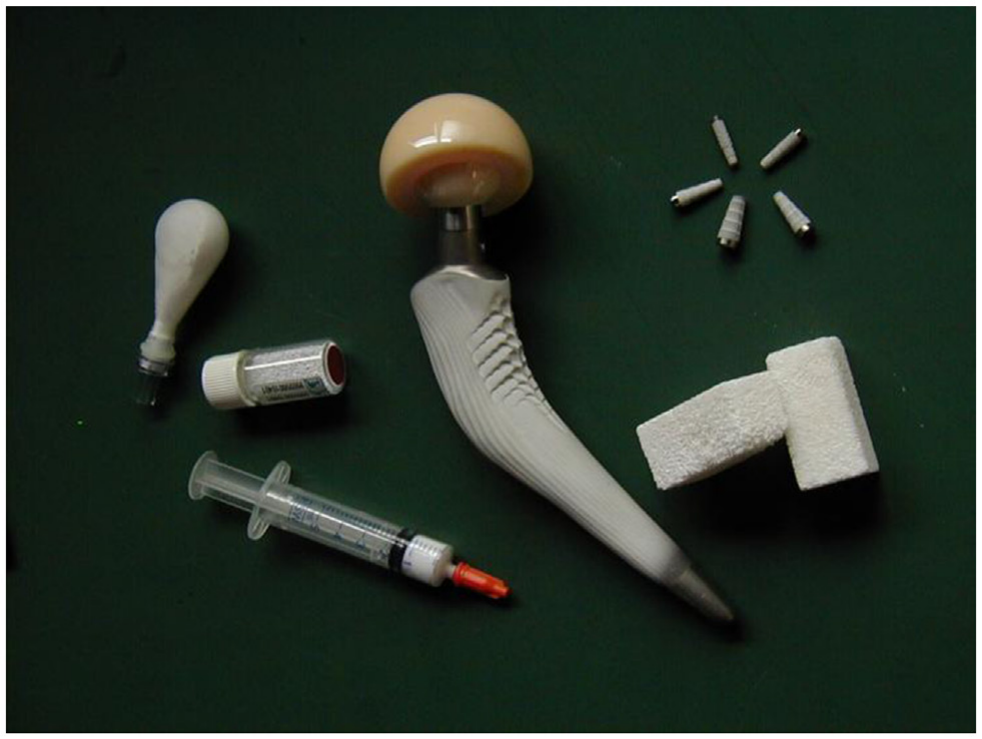
**Examples of commercial bioceramics for medical applications: powders and granules for use as bone fillers (typically calcium phosphates or bioactive glass), hemispherical acetabular cup (alumina) for hip joint prosthesis, hydroxyapatite coating on femoral metal stem, porous scaffolds (usually calcium phosphates or bioactive glass/glass–ceramic)**. Image reproduced from © Dorozhkin (2010a).

Bioceramics is a large class of specially designed ceramics for the repair and reconstruction of diseased or damaged parts of the body. Current forms of application in clinical use include solid pieces (used, for instance, in the reconstruction of middle ear ossicles or as load-bearing components of joint prostheses), powders and granules for bone filling, coatings on metal joint prostheses, injectable formulations (bone cement), and porous scaffolds (Figure 1). Based on their tissue response, bioceramics can be classified into three major families: nearly inert (e.g., alumina and zirconia), bioactive (e.g., bioactive glass), and resorbable ceramics [e.g., β- and α-tricalcium phosphate (TCP)] (Hench, 1996). Nearly, inert ceramics are generally used as femoral heads and acetabular cups for hip replacement as well as to fabricate dental implants; however, usually these materials are not used as scaffolds due to their inertness that triggers the formation of a 1- to 3-μm thick “protective” fibrous capsule on the surface of the implant. Even if there is no aggressive foreign body response, there is no bond between the implant and the host tissue (Hench, 1996).

The ability of creating a stable bond with the host tissue is of primary importance in the selection of bioceramics for making scaffolds. In this regard, bioactive as well as bioresorbable ceramics represent a valuable solution. Furthermore, the latter ones exhibit the added value of degrading gradually over a period of time while being replaced by the natural host tissue and, therefore, disappear once their task of acting as templates for new tissue has been completed (Baino and Vitale-Brovarone, 2011; Fu et al., 2011a,b).

The following sections focus on the main types of bioceramics that are currently used to fabricate scaffolds by schematically grouping the materials in their specific class from a microstructural viewpoint: crystalline ceramics, bioactive glasses, glass–ceramics, and composites. Applications and clinical developments are also shortly discussed.

### Crystalline Ceramics

The major representatives of this class are calcium phosphates that are among the most widely used crystalline ceramics for bone tissue regeneration. This is due to their exceptional properties that include (i) similarity, in terms of structure and chemical composition, to the mineral phase of bone, and (ii) osteoconductivity, i.e., the ability of providing a biocompatible interface along with bone migrates, and thus bonds to the host tissue without the formation of scar tissue (Cao and Hench, 1996; LeGeros, 2002).

Synthetic HA (Ca_10_(PO_4_)_6_(OH)_2_) has a stoichiometric calcium-to-phosphate ratio of 1:67 and, from a crystallographic point of view, is the calcium phosphate phase most similar to natural bone apatite. Because of its excellent biocompatibility and osteoconductivity, HA is successfully used as bone filler in the form of cement or granules and in the form of coatings on metallic joint prostheses. However, its use as a scaffold material is limited because of its low mechanical properties and extremely slow resorption rate (Barrere et al., 2006).

It is partly for this reason that other calcium phosphates have emerged with different degrees of solubility depending mainly on the calcium-to-phosphorous ratio (the rate of dissolution increases with decreasing Ca/P ratio) as well as on the crystallographic structure (Hench, 1996; Dorozhkin, 2007, 2010a,b). The interested reader is addressed to specific publications dealing with calcium phosphate bioceramics (Legeros et al., 2003; Dorozhkin, 2012).

A common drawback to all calcium phosphate bioceramics produced in a porous form is their low mechanical properties (brittleness, low fatigue strength) that largely limit their clinical use to non-major load-bearing parts of the skeleton. We have to consider that calcium phosphate scaffolds are often consolidated by sintering that, however, does not occur under a viscous flow regime, and thus may not lead to full densification of scaffold struts. Other materials, such as a few bioactive glass–ceramics and composites, seem to be more suitable for fabricating high-strength, tough scaffolds (Baino and Vitale-Brovarone, 2011; Fu et al., 2011a,b).

Besides calcium phosphates, alumina is another well-known example of crystalline ceramic that has been widely used for decades to fabricate components of hip and knee joint prostheses (femur head, acetabular cup, and tibial plate) primarily due to its high-strength suitable for load-bearing applications, excellent wear resistance, and bioinertness (associated with maintenance of the desired physico-chemical and mechanical properties over time) (Rahaman et al., 2007). Porous alumina is clinically used only in the fabrication of orbital implants (spherical porous scaffolds) for enucleation that should allow fibrovascular ingrowth through the pore network and remain in the patient’s anophthalmic socket indefinitely without undergoing degradation (Baino et al., 2014; Baino and Vitale-Brovarone, 2015a).

### Bioactive Glasses

It has been extensively proved that bioactive glasses are able to strongly bond to living tissues (primarily bone) creating a stable interface and to trigger a range of biological responses, such as tissue regeneration and angiogenesis while degrading over time (Hench, 2006; Jones, 2013).

These properties of bioactive glasses arise from a time-dependent modification of their surface that occurs on exposure to physiological environment. The glass surface forms a biologically active layer of HA that provides the bonding interface with host tissues, while the dissolution products (Si, Na, Ca, phosphate ions, etc.) stimulate the cells to produce new tissue (Cao and Hench, 1996).

The first bioactive glass, belonging to the 45SiO_2_–24.5Na_2_O–24.5CaO–6P_2_O_5_ (wt.%) system (45S5 Bioglass^®^), was developed by Prof. Larry Hench and coworkers in the late 1960s (Hench et al., 1971) and is in clinical use since 1985. Over the years, many other silicate, borate, and phosphate glasses have been proposed for biomedical applications, as reviewed elsewhere (Baino and Vitale-Brovarone, 2011; Rahaman et al., 2011).

Bioactive glasses are commonly produced by traditional melting-quenching routes or the sol–gel technique. Melt-derived glasses can be poured into molds to produce rods and bars or cast as components of various sizes and shapes. The melt can also be quenched in cold water to obtain a “frit,” i.e., granules and pieces of different sizes that can be easily powdered and further processed to fabricate porous scaffolds (Baino and Vitale-Brovarone, 2011). Finally, the glasses can be also spun to fabricate glass fibers that in the last decade have attracted increasing interest for application in soft-tissue engineering, especially the phosphate ones, as guides for muscle or nerve repair (Vitale-Brovarone et al., 2012a) as well as for the fabrication of glassy bone scaffolds (Gu et al., 2013). For melt-derived silicate glasses, the silica content should be <60 mol.% to allow the glass to bond with bone (Wilson et al., 1981). However, HA layer formation and bone bonding can be also achieved with glasses with up to 90 mol.% silica if the glass is obtained by a sol–gel process (Li et al., 1991). In general, sol–gel glasses were found to form a nanocrystalline HA surface layer more rapidly than melt-derived glasses due to the higher surface area available for ion-exchange phenomena (tens vs. few meter square per gram).

In the last decade, the advent of mesoporous bioactive glasses (MBGs) allowed combining superior bioactive properties (formation of a surface HA layer within few hours from contact with biological fluids) and drug uptake/release abilities in a single, multifunctional biomaterial (Arcos and Vallet-Regí, 2013).

### Glass–Ceramics

Glass can be converted by heating into a partially crystalline material containing various kinds of crystalline phases with controlled size and content depending on the thermal treatment parameters. Generally, the resulting glass–ceramic material exhibits superior mechanical properties with respect to its parent glass, specifically higher elastic modulus, hardness, failure strength, and wear resistance. Scaffolds are often produced by sintering, which requires glasses to be heated above their glass transition temperature in order to initiate localized flow. Many bioactive glasses, including 45S5 Bioglass^®^, crystallize immediately above their glass transition temperature; therefore, sintered bioactive glass scaffolds are often glass–ceramic scaffolds (Gerhardt and Boccaccini, 2010; Baino and Vitale-Brovarone, 2011).

45S5 Bioglass^®^-derived scaffolds suffer from some drawbacks as the base glass tends to crystallize before full densification is achieved (sintering end), thereby originating extremely brittle glass–ceramic porous products; furthermore, scaffold bioactivity seems to be partially suppressed by the development of a sodium–calcium–silicate crystalline phase (Chen et al., 2006). In the attempt to overcome these drawbacks, interesting results have been obtained by various research groups that proposed alternative glass–ceramics. For instance, Vitale-Brovarone et al. (2007) used the bioactive glass CEL2 (45SiO_2_–26CaO–15Na_2_O–3P_2_O_5_–4K_2_O–7MgO mol.%) to fabricate foam-like glass–ceramic scaffolds exhibiting compressive strength up to 1 MPa (porosity 70 vol.%) and an excellent biological compatibility with osteoblasts; more recently, the same research group successfully optimized the process parameters to obtain scaffolds with higher strength (5–6 MPa) within the typical range of cancellous bone (2–12 MPa) (Vitale-Brovarone et al., 2009; Baino et al., 2013). Glass–ceramic bone-like scaffolds based on the experimental glass SCNA (57SiO_2_–34CaO–6Na_2_O–3Al_2_O_3_ mol.%) can reach a compressive strength of 15 MPa (porosity around 65 vol.%), which makes them suitable for load-bearing applications but retain an extremely moderate bioactivity (Vitale-Brovarone et al., 2012b; Baino and Vitale-Brovarone, 2014).

### Composites

A crucial aspect for the success of scaffolds in tissue engineering and regeneration of tissues is that the structure and properties of the scaffolds must be pertinent to the tissue concerned and the mechanical loads that it will experience *in vivo*. Like most ceramic materials, bioceramics have the disadvantage of exhibiting low fracture toughness (i.e., brittleness) and this could limit their use in load-bearing applications. Furthermore, their high stiffness may restrict the use of bioceramics in non-osseous applications, where adequate compliance with soft tissues is necessary (Miguez-Pacheco et al., 2015a).

One approach that aims to overcome these problems is the combination of bioceramics with polymers to produce a composite scaffold, which makes the most of both materials. Typically, bioceramics are added as fillers or coatings to the polymer matrix to improve its mechanical proprieties, i.e., to increase strength and stiffness as well as to effectively induce enhanced bioactivity (Mohamad Yunos et al., 2008).

Following an alternative strategy, Bretcanu et al. (2007) fabricated porous composites by using a bioceramic scaffold (45S5 Bioglass^®^) as a porous inorganic matrix and by coating it with poly(3-hydroxybutyrate) (P3HB). The polymer was specifically introduced to strengthen the 45S5 Bioglass^®^ scaffold structure, in fact, the P3HB layer acted as a glue, thereby holding the inorganic particles together when the scaffold struts started to fail. The compressive strength of such a composite scaffold (up to 1.5 MPa) was twice than that of bare 45S5 Bioglass^®^ scaffolds (up to 0.4 MPa) (Chen et al., 2006).

Added values, such as drug uptake/release, are also provided to the composite if mesoporous glass particles are used as a second phase (Arcos and Vallet-Regí, 2013).

Both non-degradable and degradable polymers have been used in the fabrication of composite scaffolds; however, stable polymers often have low biocompatibility as they tend to become surrounded by a fibrous capsule once implanted. Therefore, there have been several attempts to create composites based on the combination of biodegradable polymers and bioceramics. The first composites investigated were comprised of HA or TCP used as inorganic phases while poly(l-lactic acid) (PLLA), poly(d/l-lactic acid) (PDLLA), poly(glycolic acid) (PGA), and their copolymers (PLGA) as organic ones (Ambrosio et al., 2001; Deng et al., 2001; Kasaga et al., 2001; Xu et al., 2004). HA/polyethylene porous composites, marketed under the commercial name “Hapex,” are currently used in the clinical practice for the repair of orbital floor fractures (Tanner, 2010).

More recently, attention has moved toward nano-bioceramic/polymer composites, which have the potential to improve interaction with the host tissue/cells (Erol-Taygun et al., 2013). In this regard, one of the most fascinating challenges is to develop smart composite biomaterials with nanoscale interaction between the bioactive inorganic phase and the organic one, so that the scaffold could degrade as a single material rather than having mismatched degradation rates of glass and polymer phase. As recently underlined by Jones (2009), this intimate interaction should allow cells to come into contact with both phases at one time, and the scaffold should degrade at a single rate.

A special mention should be devoted to the so-called “star gels,” which are a particular type of organically modified silicates (“ormosils”) having an organic core surrounded by flexible arms that are terminated in alkoxysilane groups able to form a silica-like network during the sol–gel process (Vallet-Regí et al., 2006). These hybrid materials show bioactive properties and have fracture toughness higher than that of sol–gel glasses and comparable to that of cancellous bone, thus having promise for tissue engineering applications that require good long-term fatigue behavior (Manzano et al., 2006).

Fabrication of bioceramic/metal composites has been also reported where the ceramic phase is applied in the form of a coating. Metallic materials, such as stainless steel, titanium, and Co–Cr–Mo alloy, have become the materials of choice for load-bearing prostheses due to high-strength, good fatigue resistance, and favorable machining properties. Some metallic materials, however, may produce adverse effects such as the release of significant amounts of metal ions into the tissues, which may result in complications, such as inflammatory and immune reactions (Alvarez and Nakajima, 2009). Thus, there is a need to further improve the biocompatibility between metallic materials and host tissue (primarily bone). Wang et al. (2009) prepared porous TiNbZr alloy scaffold coated with calcium phosphate to improve osteoconductivity. Cell culture experiments showed that the surface-modified TiNbZr scaffolds were more favorable for the adhesion and proliferation of osteoblast-like cells compared to bare metal scaffolds.

## Fabrication Technologies of Bioceramic Scaffolds

A wide range of processing routes has been proposed for the production of bioceramic porous scaffolds for tissue engineering. These include techniques developed *ad hoc* or often adapted from other contexts, such as foaming, solid freeform fabrication (SFF), starch consolidation, organic phase burning-out, and sponge replication.

These various methods provide a mean to control the 3-D structure of tissue engineering constructs, and processing can strongly influence various characteristics of the scaffold. Each method, in fact, is best suited for producing a specific range of pore size and distribution, interconnectivity and overall porosity in addition to strut thickness and orientation. Thus, the most appropriate technique must be accurately selected to meet the demands of the specific type of tissue (Colombo, 2006).

Moreover, each fabrication method differs in terms of overall cost, making some of them attractive for a large-scale production, while others are more appropriate for the development of value-added products.

The following sections examine the main available techniques to fabricate bioceramic scaffolds, highlighting time by time the merits and drawbacks of each method. A comparison of these techniques is given in Table 2. An overview of the main methods used to fabricate bioceramic containing composite scaffolds is also provided.

**Table 2:** **Comparison of different techniques (listed in alphabetical order) for the fabrication of bioceramic scaffolds (non-composite) on the basis of their advantages and disadvantages**.

Technique	Advantages	Disadvantages	Reference
Foaming methods (general)	Allows manufacturing of both closed and open-cell foams; good versatility of final part shapes, as the solution can be cast in molds without additional machining	Difficulty in achieving high interconnectivity; non-porous external surface	Jones and Hench (2003) and Colombo (2006)
H_2_O_2_ foaming	Simple	Low porosity control laminar pore structure with poor 3-D interconnection	Li et al. (2002) and Navarro et al. (2004)
Sol–gel foaming	Hierarchical structure can be obtained (macroporous scaffold combined with ordered mesoporous texture)	Need for a high degree of control of the foam	Akkus et al. (2002) and Jones and Hench (2004)
*In situ* polymerization of organic monomer (gel-cast foaming)	Highly porous ceramic; high-strength properties due to the less flawed structure and dense struts and walls produced	Low pore interconnectivity	Sepulveda and Binner (1999), Ortega et al. (2002), Ramay and Zhang (2003), and Wu et al. (2011b)
Organic phase burning-out/space holder	High mechanical strength	Difficult to obtain a homogeneous distribution of pores; poor interconnectivity	Baino et al. (2009) and Wu et al. (2009)
Solid freeform fabrication (SFF) (general)	Customized objects; reproducible	Costly; resolution needs to be improved to the micro-scale	Hollister (2005)
SLA	Complex internal features can be obtained	Only applicable using ceramic/photopolymer blends	Levy et al. (1997), Tesavibul et al. (2012), Scalera et al. (2014), and Sabree et al. (2015)
SLS	High accuracy; good mechanical strength; a broad range of materials can be processed	High temperatures during process; trapped powder is difficult to remove	Hutmacher et al. (2004)
3-D printing	Fast processing; no toxic components; water used as a binder; tunable mechanical properties	Trapped powder issue	Yun et al. (2007), Fu et al. (2011a,b), Garcia et al. (2011), Wu et al. (2011a), Bose et al. (2013), and Liu et al. (2013)
Sponge replication	Reticulated open-cell material; applicable to any ceramic material that can be dispersed into a suspension; no toxic chemicals needed	Mechanical properties might be poor	Chen et al. (2006), Zhu et al. (2008), Vitale-Brovarone et al. (2009), Zhu and Kaskel (2009), Wu et al. (2010), Baino et al. (2013), and Baino and Vitale-Brovarone (2014)
Starch consolidation	Environment-friendly; low-cost	Pores might be poorly interconnected	Lyckfeldt and Ferreira (1998) and Vitale-Brovarone et al. (2004, 2005)
Thermal bonding of short glass fibers	Simple; no need for any additional material except fibers and mold; glassy scaffolds can be obtained	Mechanical properties might be poor	Pirhonen et al. (2003), Moimas et al. (2006), Gu et al. (2013), and Tirkkonen et al. (2013)

### Foaming Methods

Highly porous ceramics can be produced by dispersing a gas in the form of bubbles into a ceramic suspension or colloidal sols, followed by solidification, to obtain pores in the range of 20 μm up to 1–2 mm (Jones and Hench, 2003). The various foaming techniques developed in the literature are based on two approaches: (i) incorporating an external gas by mechanical frothing, injection of a stream of gas, or introduction of an aerosol propellant and (ii) evolution of a gas *in situ*. The decisive step in direct foaming methods is the stabilization and setting of the wet foams. These, in fact, need to be set in order to maintain their porous morphology before heating at high temperature for sintering/ceramization. Furthermore, several transformations in the bubble structure might occur within the interval between foam generation and foam solidification. For instance, the gas bubbles initially have a spherical shape (nucleation phase) and later grow as polyhedral cells (Colombo, 2006). In order to retain the cellular morphology and prevent the collapse of the foamed structure, surfactants are generally used to stabilize the bubbles formed in the liquid phase as they reduce the surface tension of the gas–liquid interfaces. Surfactants stabilize the system for a limited period of time; hence, a further mechanism is then required to provide a more permanent form of stabilization (Sepulveda and Binner, 1999).

The incorporation of bubbles can be brought about by a variety of processing routes.

H_2_O_2_ foaming involves mixing ceramic powder with an aqueous solution of H_2_O_2_ as a foaming agent; then, the resulting mixture is cast into molds and stored into an oven at 60°C. At this temperature, H_2_O_2_ decomposes and the oxygen released tends to form bubbles in the slurry and, thus, gives rise to the foaming process. The sample is then sintered to obtain crystalline ceramics, bioactive glass, and calcium phosphate scaffolds depending on the initial powders (Navarro et al., 2004). By varying the amount of H_2_O_2_ incorporated and the thermal treatment, the percentage of porosity and pore size can be modulated. However, an intrinsic shortcoming of this foaming method is that pores are interconnected only in a laminar manner, resulting in poor interconnection in the direction perpendicular to the laminae (Li et al., 2002).

**Figure 2:**
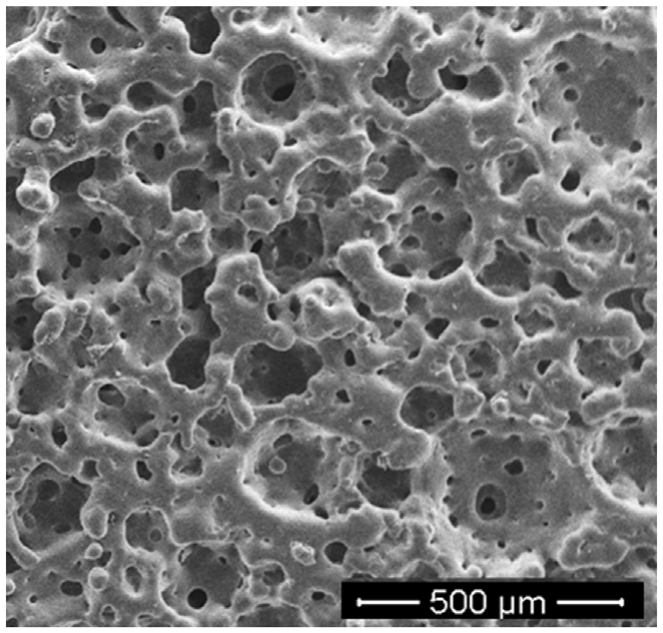
**Bioactive glass–ceramic scaffold obtained by gel-cast foaming followed by a freeze–drying step before sintering (courtesy of Giorgia Novajra)**.

An alternative to H_2_O_2_ foaming is *in situ* polymerization of an organic monomer (or gel-cast foaming). A high-solid-load aqueous ceramic suspension is prepared that also incorporates an organic monomer, which must be soluble in water (e.g., acrylates) together with an initiator and a catalyst to provide *in situ* polymerization (Ortega et al., 2002; Wu et al., 2011b). The two latter ingredients are necessary to control the actual beginning of the polymerization reaction (i.e., the induction time – period of inactivity between the addition of reagents and the polymerization reaction onset), which, in the processing of porous ceramics, must take place during casting. After the addition of a foaming agent (surfactant), the suspension is mechanically agitated to obtain a wet ceramic foam. The foam is cast into the appropriate mold and, after polymerization is complete, the green body is strong enough to be removed from the mold and transferred to an oven for drying, burning-out of the polymer, and sintering of ceramic particles. The resulting ceramic foam exhibits higher strength magnitudes compared to other conventional methods due to the less flawed structure (low amount and size of the defects) and dense struts and walls produced. However, the porous structure results poorly interconnected and non-homogeneous (Sepulveda and Binner, 1999). The final cell size distribution and strut thickness can be engineered during processing by controlling enlargement of bubbles and thinning of lamellas (cell walls) upon the induction period. This can be efficiently done by altering the concentration of the initiator and the catalyst; however, other parameters such as temperature and pH have also been found to play a significant role (Sepulveda and Binner, 1999). Another advantage of this technique is the ability to produce porous scaffolds with a high degree of complexity. The casting process, in fact, allows shaping forms/profiles without the need of machining. Additionally, if further details are required, the dried green foams are strong enough to withstand machining (Colombo, 2006). Gel-cast foaming has also been combined with the foam replica method (the latter described in Section “Sponge Replica Method”) to produce HA scaffolds with interconnected pores (Ramay and Zhang, 2003). Gel-cast foaming can also involve the use of gelling polymers (e.g., gelatin) with no need for initiator and catalyst (e.g., gelation can take place with a decrease in temperature); in this case, a supplementary freeze–drying step before sintering is necessary (Novajra et al., 2015a,b). The structure of a scaffold produced by gel-cast foaming is shown in Figure 2.

A third option is sol–gel foaming, a process that combines sol–gel technology – a chemical-based wet synthesis route, which involves the conversion of a solution containing ceramic precursors (sol) into a network of covalently bonded silica via inorganic polymerization reactions – and mechanical frothing (Akkus et al., 2002). After heat treatment, a glass or glass–ceramic construct can be obtained exhibiting a hierarchical structure with interconnected macropores for tissue ingrowth (10–500 μm) and a mesoporous texture (channels in the 2–50 nm range) that promotes cell adhesion and adsorption of biological metabolites while intensifying the rate of surface reactions *in vitro* and *in vivo* (especially the formation of surface HA layer) (Jones and Hench, 2004). The latter feature is tuned by including in the sol a surfactant that acts as a template for supramolecular self-assembly; this process is also referred to as evaporation-induced self-assembly (EISA) (Brinker et al., 1999). The steps involved in the process are (1) preparation of a sol from a mixture of distilled water, appropriate precursors (metal alkoxides, such as tetraethylorthosilicate and triethylphosphate), salts (CaNO_3_), and a hydrolysis catalyst (dilute acid), (2) foaming by vigorous agitation with the addition of a gelling agent, a surfactant, and distilled water, (3) casting of foamed mixture into molds, (4) aging to achieve gelation of the sol, (5) removal of the solvent by drying at low temperature, and (6) sintering to obtain porous components. Highly bioactive bone-like 3-D scaffolds can be successfully obtained by this method (Jones and Hench, 2004); an unavoidable limitation is the intrinsic brittleness of the porous product due to the nanoporous texture, which poses critical issues in view of the safe implantation of the device (too low mechanical properties) (Baino and Vitale-Brovarone, 2011).

### Starch Consolidation

This method uses corn-, rice-, or potato-derived starch granules both as a pore former and a binder to fabricate porous ceramics. The main advantages of this processing technique are its low cost and its environment-friendly nature.

The process involves mixing of starch granules, ceramic powder, and distilled water to obtain a suspension that is continuously stirred and maintained at 60–80°C. In this temperature range, starch undergoes swelling due to water absorption, leading to a gel-like material that, after consolidation, is thermally treated to burn-out the organic phase and to sinter the ceramic matrix. Low dimensional changes occur during consolidation and drying, which ease the control of the ultimate dimensions of the component after sintering (Lyckfeldt and Ferreira, 1998).

Historically, this method was one of the first used to process bioactive glasses in a porous form (Vitale-Brovarone et al., 2004, 2005); albeit the mechanical properties of the resulting glass–ceramic scaffolds (compressive strength about 6 MPa) were comparable to those of cancellous bone (2–12 MPa), the porosity was too low (40 vol.%) and poorly interconnected for deeming an eventual clinical application. Therefore, other polymer phases (apart from starch) have been experimented as a pore former for tissue engineering bioactive glass scaffolds.

### Organic Phase Burning-Out

The organic phase burning-out (or space-holder method) is another strategy for producing porous scaffolds. In this method, ceramic powders are mixed together with a solid polymeric phase of synthetic [e.g., poly(methyl methacrylate) or polyethylene microbeads] (Baino et al., 2009) or natural origin (e.g., rice husk) (Wu et al., 2009). Afterwards, the blend is pressed to obtain a “green body” and thermally treated at high temperature. Upon heating, the polymeric particles that fill in the space within the volume of the component decompose, whereas the inorganic particles sinter, leading to a porous body displaying a negative replica of the original sacrificial template (Colombo, 2006; Baino and Vitale-Brovarone, 2011). Since sintering requires higher temperatures than pyrolysis, the ceramic matrix has to be partially consolidated before removal of the sacrificial material, so that the porous structure does not collapse during the polymer removal step; therefore, binders are generally incorporated in the mixture (Studart et al., 2006).

Both closed and open cell ceramic foams can be obtained, depending on the volume fraction and nature (significantly affecting the amount of gas developed during burning-out) of the sacrificial polymer. Nevertheless, pore interconnectivity is generally low due to the difficulty in maintaining a homogeneous distribution of the polymer spheres (Baino et al., 2009; Wu et al., 2009). Due to the presence of thick, dense struts, scaffolds produced by this method can exhibit high mechanical strength, even comparable to that of cortical bone (Baino et al., 2009).

In order to attain a highly porous structure, a large proportion of the polymeric phase in the starting mixture is necessary. This typically causes the development of a large amount of gas during heating that can cause the formation of cracks in the ceramic body (Bretcanu et al., 2014). Thus, the process needs to be attentively controlled to avoid the formation of defects in the final component.

### Sponge Replica Method

**Figure 3:**
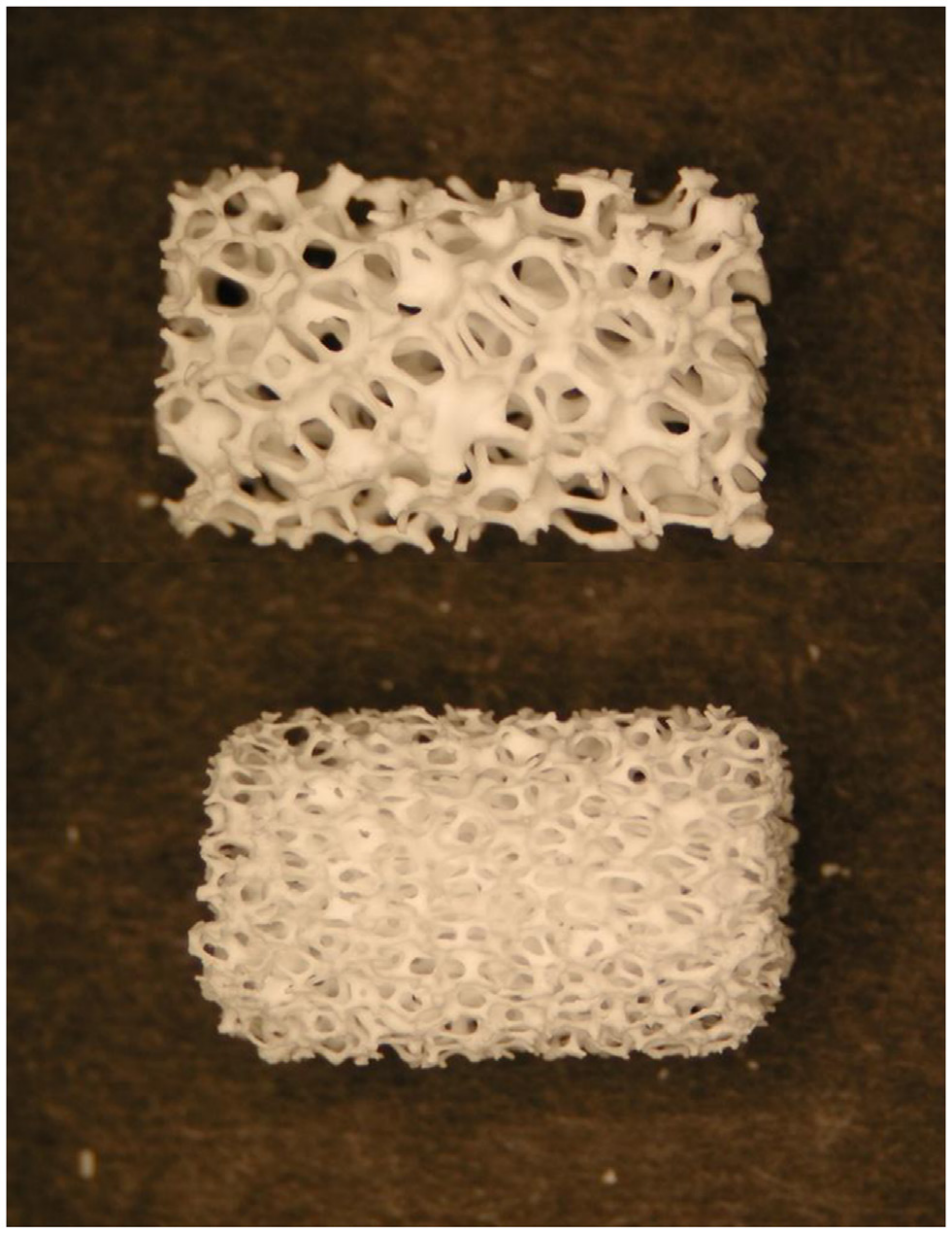
**Photographs of two commercially available hydroxyapatite cylindrical scaffolds with different porosity produced by sponge replica method**. The length of the scaffolds is about 15 mm. Image adapted from © Dorozhkin (2010a).

The sponge replication method was patented by Schwartzwalder and Somers (1963) and, since then, it has become the most popular and effective method of producing foam-like ceramic scaffolds for tissue engineering. This success is primarily attributed to the simplicity and flexibility of the method, as it is applicable to any ceramic material that can be appropriately dispersed into a suspension. It has been observed that the reticulated open-cell structure (i.e., consisting of interconnected voids surrounded by a web of ceramic ligaments, the struts) that can be obtained using the foam replica method is the most suitable for bone tissue engineering scaffolds (Table 1) as it closely mimics the 3-D trabecular architecture of cancellous bone (Figure 3). Another key strength of this method is that the starting sponge can be easily cut and conformed to match the size and shape of the tissue defect, so that – at least ideally – personalized scaffolds could be fabricated according to the patient’s clinical needs (Vitale-Brovarone et al., 2009).

This process involves the impregnation of an open-cell porous template of synthetic (typically a polyurethane sponge) or natural material (e.g., marine sponge) with a slurry of finely divided ceramic powder and a binding agent [e.g., poly(vinyl alcohol), colloidal silica]. The sponge is then squeezed to remove the excess slurry and enable the coating of the sponge struts with a thin layer of the slurry. After drying, the coated template is pyrolyzed while the remaining ceramic coating is sintered at higher temperatures to obtain a porous ceramic exhibiting the same architecture as the sacrificial template (positive replica). Therefore, the morphological characteristics of the ceramic foam are directly related to those of the polymeric template used (Schwartzwalder and Somers, 1963).

The most crucial step in the process is the production of a uniform coating on the polymeric structure. In more detail, the affecting factors are (1) the rheology of the impregnating suspension and (2) its adhesion on the struts of the polymeric sponge. The suspension should be sufficiently fluid to allow penetration into the cells of the sponge upon compression and expansion, but viscous enough to avoid drainage of the remaining coating. It is also worth mentioning that incomplete removal of the excess slurry leads to a structure with a certain degree of closed porosity (Schwartzwalder and Somers, 1963; Colombo, 2006).

The sponge replica method has been recently applied in combination with EISA method to produce hierarchical porous bioactive glass scaffolds, where a polyurethane foam and a surfactant were used as co-templates for scaffold macropores and mesopores, respectively (Zhu et al., 2008; Zhu and Kaskel, 2009). These scaffolds are highly bioactive but exhibit dramatic brittleness due to the presence of the mesoporous texture. Wu et al. (2010) tried to improve the mechanical properties of these hierarchical porous constructs by depositing a silk coating on the strut, but their compressive strength still remained too low (few hundreds of kilopascal) for deeming a safe clinical application.

### Solid Freeform Fabrication

Solid freeform fabrication, also referred to as rapid prototyping, denotes a set of emerging moldless techniques that use layer-wise manufacturing strategies to create scaffolds with customized external shape and pre-designed internal architecture (strut features, pore arrangement, size, and distribution) directly from a computer-generated 3-D model. This model is a 3-D reconstruction of the patient-specific tissue defect, which can be acquired from patient’s computed tomography data or magnetic resonance imaging. Further details regarding the micro-environment can be developed by making use of computer-aided design (CAD). One of the main advantages of SFF technology is the ability to fabricate components with highly reproducible architecture and compositional variation (Hutmacher et al., 2004). This set of techniques is particularly valuable to produce functionally graded bioceramic and composites (Miao and Sun, 2010).

A number of SFF strategies have been adopted to manufacture scaffolds for tissue engineering applications (Hollister, 2005).

**Figure 4:**
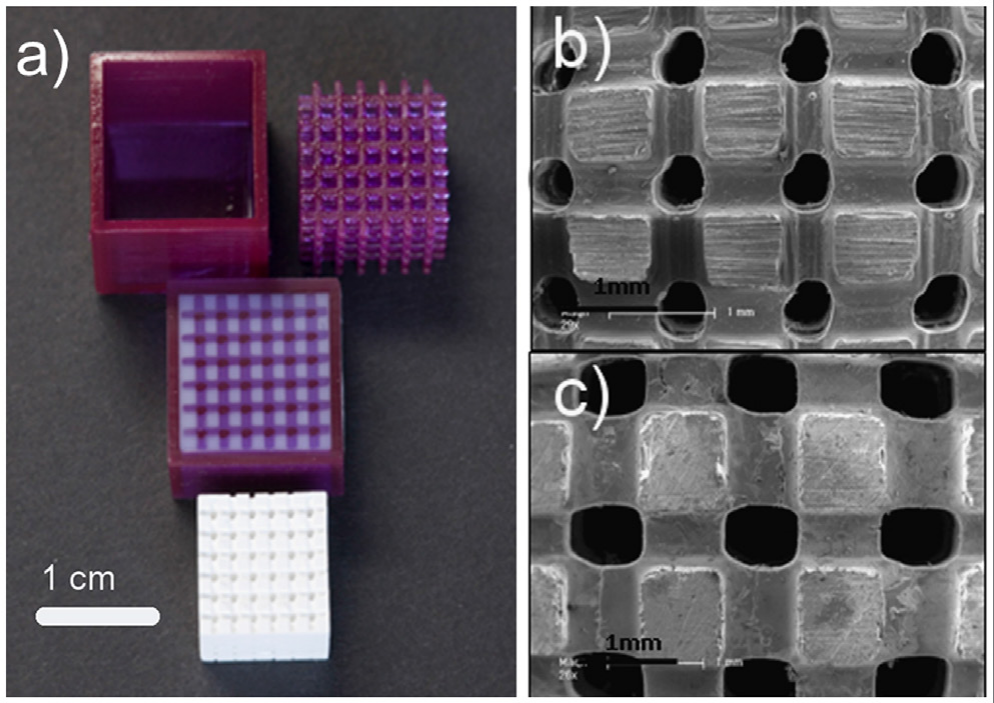
**Wollastonite containing glass–ceramic scaffolds produced by stereolithography: (A) original mold fabricated by stereolithography, filled mold and final sintered scaffold structure showing shrinkage after sintering (1200°C); (B,C) SEM images showing a general view of the scaffold structure and morphology (nominal pore size of 400 and 500 μm, respectively)**. Images adapted from © Sabree et al. (2015).

Stereolitography (SLA) uses a blend of ceramic powders and a photocurable monomer. A UV laser beam, which cures the monomer, is selectively scanned over the surface of the blend following the cross-sectional profiles of the CAD model; subsequent layers are built directly on top of previously cured layers with new layers of blend being deposited. After this step, the material not cured by the laser can be drained away and sintering is performed to produce the final object (Levy et al., 1997; Hutmacher et al., 2004). Fabrication of HA and amorphous calcium phosphate scaffolds for hard tissue repair using SLA has been extensively reported in the literature (Hollister, 2005; Scalera et al., 2014). Recently, Tesavibul et al. (2012) proposed the use of a lithographic method to fabricate 45S5 Bioglass^®^-derived scaffolds with highly ordered pore arrangement. Stereolitographic fabrication of wollastonite containing glass–ceramic scaffolds with high-strength properties was also reported by Sabree et al. (2015) (Figure 4).

**Figure 5:**
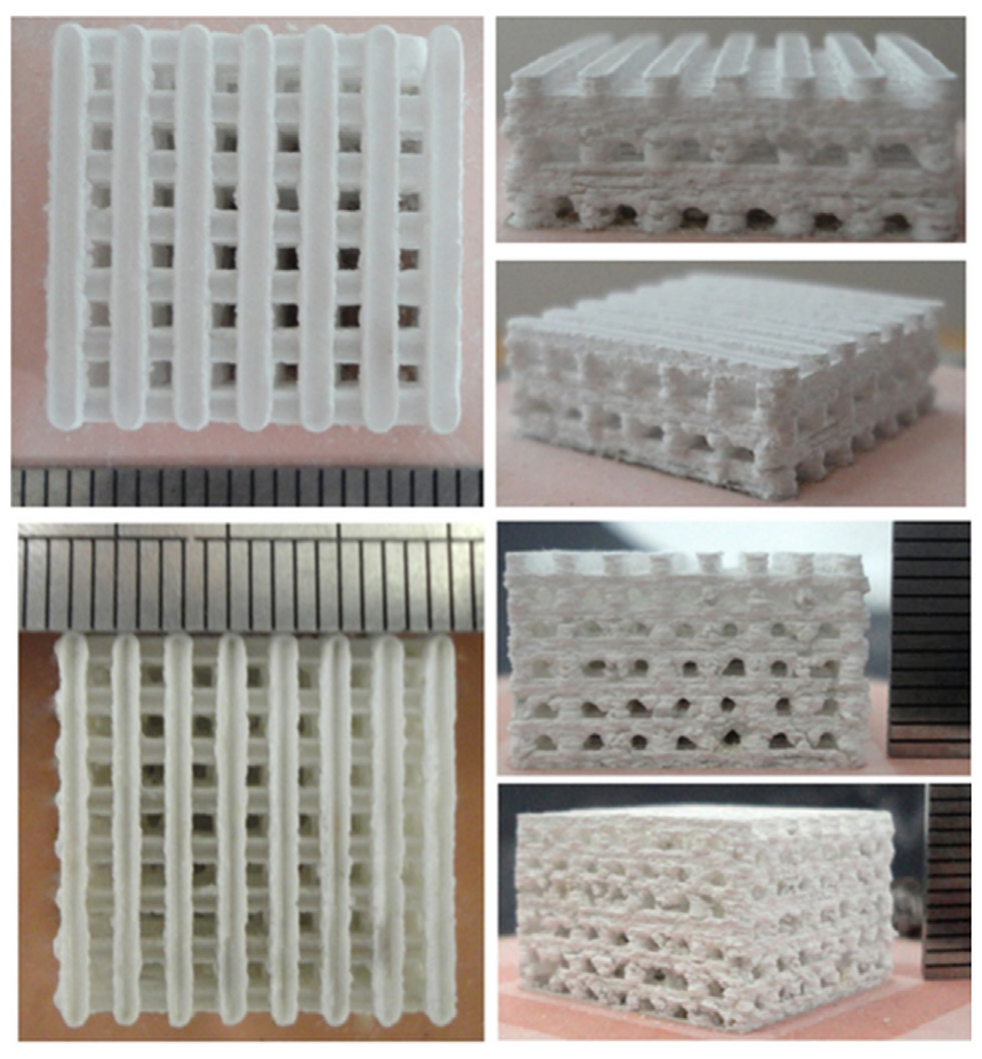
**Calcium phosphate (HA/β-TCP = 60/40 wt.%/wt.%) scaffolds fabricated by selective laser sintering: the scaffold architecture in 3-D is built up layer by layer**. Images adapted from © Gao et al. (2014).

Selective laser sintering (SLS) is a technique that employs a CO_2_ laser beam to sinter thin layers of powdered ceramic materials to form 3-D objects. The laser beam is scanned over the powder bed following CAD data, thus raising the temperature of powders only in selected areas. In this way, particles fuse together and subsequent layers can be built directly on the top of the previously sintered material. Scaffolds from nano-HA and β-TCP as well as ceramic/polymer composites have been prepared using SLS technology (Hutmacher et al., 2004). Gao et al. (2014) recently reported the fabrication of biphasic calcium phosphate scaffolds by SLS, too (Figure 5).

**Figure 6:**
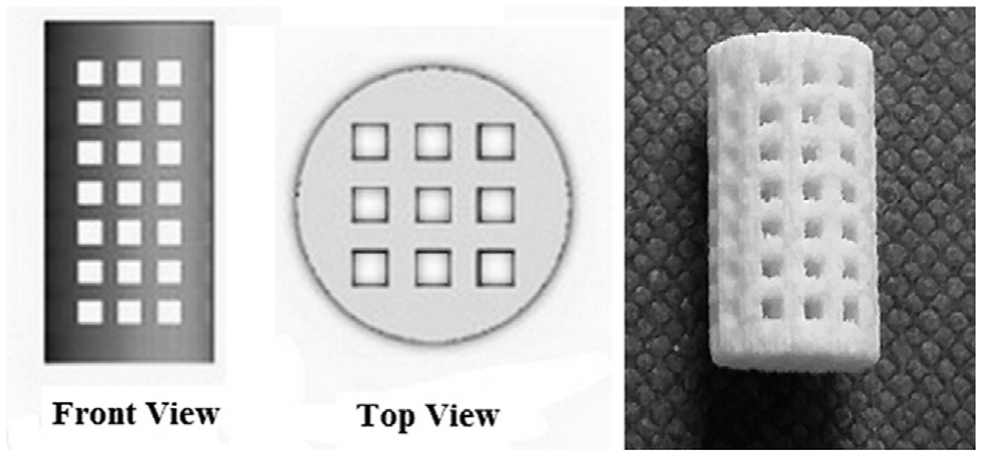
**3-D printing of calcium sulfate cylindrical scaffolds: scaffold design (front and top views) using SolidWorks and 3-D printed specimen (sample length 12 mm)**. Image reproduced from © Farzadi et al. (2015).

A third very valuable option to produce porous ceramic scaffolds is 3-D printing (3DP), developed in the early 1990s at MIT. 3DP is a powder-based technology that employs a printer head – which moves in accordance to the object profile being generated by a computer system – to eject and deposit binder onto the powder surface and bonds the granules in the selected regions. Subsequently, a fresh layer of powder is laid down by a set of rollers. The cycle continues until the whole object is completed and at this point an airflow is used to remove unbound powder. The objects are sintered at high temperatures to achieve sufficient strength of the bodies and to remove the binder safely. Binders can be either organic (e.g., starch based) or water based. A wide variety of ceramic materials for tissue engineering have been processed using 3DP, such as HA, calcium phosphates, calcium sulfate, bioactive glasses, and ceramic composites, with a regular 3-D architecture and pore arrangement (Figure 6); however, extensive optimization is needed to process good quality parts with 3DP for any new material/composition, which is – together with the quite high cost of instrumentation – the major drawback of this approach (Bose et al., 2013).

Methods referred to as robocasting and direct ink-write assembling belong to the broad class of 3DP techniques. In this regard, bioactive glass scaffolds with a regular arrangement of pores in 3-D and extraordinarily high mechanical performances in compression and flexure were proposed for possible application in the substitution of cortical bone and load-bearing segments of the skeleton (Fu et al., 2011a,b; Liu et al., 2013).

3-D printing has also been applied to fabricate MBG scaffolds. Yun et al. (2007) and Garcia et al. (2011) prepared hierarchical 3-D porous MBG scaffolds using a combination of double polymer template and rapid prototyping techniques. In their study, they mixed MBG gel with methylcellulose and then printed, sintered at 500–700°C to remove polymer templates and obtained MBG scaffolds. Although the obtained MBG scaffolds have uniform pore structure, their mechanical strength is compromised because of the incorporation of methylcellulose, which results in some micropores. Recently, Wu et al. (2011a) reported a new facile method to prepare hierarchical and multi-functional MBG scaffolds with controllable pore architecture, excellent mechanical strength, and mineralization ability for bone regeneration by a modified 3DP technique using poly(vinyl alcohol) as a binder. The obtained 3DP MBG scaffolds possess a compressive mechanical strength (16 MPa), which is about 200 times that of the MBG scaffolds prepared using a traditional polyurethane foam as a template.

### Thermal Bonding of Short Glass Fibers

Porous 3-D scaffolds can also be obtained using glass fibers as a starting material. The fibers, with diameters typically ranging from tens to few hundreds of micrometers, are cut and disposed into a mold in a random arrangement with porosity originating from the free space between them. Then, a thermal treatment allows this porous structure to be stabilized by thermally bonding (sintering) the glass fibers in order to obtain glass scaffolds. The scaffolds produced by this method show a high degree of pore interconnectivity and the final scaffold structure can be tailored acting on the fiber size, the sintering time, and temperature (Pirhonen et al., 2003; Moimas et al., 2006).

**Figure 7:**
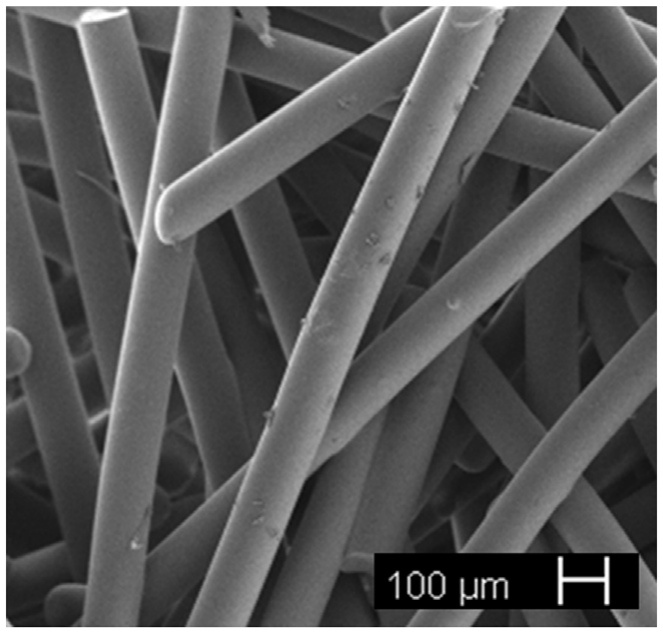
**Resorbable glass fibrous scaffold obtained by thermal bonding of short glass fibers (courtesy of Giorgia Novajra)**.

Since 45S5 Bioglass^®^ is not easy to draw into fibers without devitrification due to its narrow working range, other glass formulations that can be easily spun have been proposed in the literature for fibrous scaffold production, in particular, silicate (e.g., 13–93, 9–93) and borate bioactive glasses (e.g., 13–99B3) as well as mixtures of them (Gu et al., 2013). A porous scaffold made of glass fibers with nominal composition 11.1–12.0 Na_2_O, 15.0–17.1 K_2_⋅O, 2.8–3.3 MgO, 12.7–15.2 CaO, 2.7–3.8 P_2_O_5_, 1.0–1.4 B_2_O_3_, 0.0–0.6 TiO_2_, and 48.5–52.0 SiO_2_ wt.% (Tirkkonen et al., 2013) is currently available on the market (Inion BioRestore™, Inion Oy, Tampere, Finland) as a graft material (porous morsels) for bone defect restoration. Research studies on thermally bonded phosphate glass fibrous scaffolds are currently ongoing (Figure 7).

### Processing Technologies for Bioceramic Containing Composite Scaffolds

Numerous fabrication techniques have been described to produce 3-D porous bioceramic/polymer composite scaffolds, including space-holder, gas foaming, thermally induced phase separation (TIPS), and SFF. These methods have been extensively reviewed in the literature (Rezwan et al., 2006; Mohamad Yunos et al., 2008). Among all, TIPS can be considered the technique of choice if scaffolds with highly oriented porosity need to be prepared. This pore structure differs considerably from the isotropic structure and equiaxed pores that are typically obtained by the “conventional” methods. The TIPS process has been widely used to produce composite scaffolds based on PLGA and PDLLA foams containing 45S5 Bioglass^®^ particles as bioactive inclusions (Maquet et al., 2003, 2004; Verrier et al., 2004; Blacher et al., 2005).

Polymer-coated bioceramic scaffolds can be produced by a dipping method that involves the dipping of a bioceramic scaffold in a polymer solution followed by drying in air (Bretcanu et al., 2007); the polymer coating is useful to improve the mechanical properties of the scaffold, especially the fracture toughness (Rehorek et al., 2013).

A dip-coating approach has been also reported to apply a biomimetic calcium phosphate layer on metal scaffolds (TiNbZr alloy) to improve their biocompatibility (Wang et al., 2009).

A highly versatile and promising approach to produce bioceramic scaffolds coated with different materials (polymers, other ceramic phases) is the electrophoretic deposition (EPD), which uses the electrophoresis mechanism for the movement of charged particles suspended in a solution under an electric field, in order to deposit them in an ordered manner on a substrate to develop thin and thick films and coatings (Boccaccini et al., 2010). Fiorilli et al. (2015) reported the successful EPD of MBG onto a strong, nearly inert glass–ceramic scaffold to obtain a bioactive high-strength construct for load-bearing applications in bone tissue engineering. The use of EPD to produce carbon nanotube (CNT) coatings for smart applications in tissue engineering has been also investigated, for example, Meng et al. (2011) incorporated CNTs into 45S5 Bioglass^®^-derived glass–ceramic scaffolds by EPD and cultured mesenchymal stem cells on the constructs with and without electrical stimulation, and they observed that the electrical conductivity associated with the CNTs can promote the proliferation and differentiation of the cells attached onto the scaffold.

## Clinical Applications of Bioceramic Scaffolds: Present and Future

An overview of the applications of bioceramic scaffolds in medicine is summarized in Table 3. As a result of biomechanical limitations, bioactive glasses, glass–ceramics, and calcium phosphates are mainly used in low-/non-load-bearing applications or compressive load situations in solid or powder form, such as bone restoration and augmentation, middle ear repair, vertebral, and iliac crest replacements (Hench, 1998; Dorozhkin, 2010a). Thermal-sprayed HA coatings on metal joint prostheses are also used in the clinical practice by surgeons (Sun et al., 2001). Bioactive glass–ceramic porous coatings on alumina acetabular cups have been recently proposed to improve osteointegration of prosthetic devices (Vitale-Brovarone et al., 2012b; Baino et al., 2015, 2016a).

**Table 3:** **Applications of bioceramic scaffolds in tissue engineering**.

Field of application	Material/scaffold involved	Recipient	Stage of use/research	Reference
Bone defect repair	Porous scaffolds made of HA, biphasic calcium phosphates, bioactive glasses (e.g., 45S5 Bioglass^®^, 13–93)	H	Clinical use (the products are FDA approved)	Hench (1998) and Dorozhkin (2010a, 2012)
Joint prosthesis	Bioactive glass–ceramic coating with trabecular architecture on bioceramic acetabular cup	–	Promising experimental results achieved in the framework of the EC-funded project “MATCh.” Neither *in vitro* nor *in vivo* tests are currently available in the literature	Vitale-Brovarone et al. (2012a,b) and Baino et al. (2015, 2016a,b)
Orbital implant	Porous spheres made of alumina (the so-called “Bioceramic implant”), HA (examples of commercial products: coralline HA – Bioeye^®^, synthetic HA – FCI3, bovine HA – Molteno M-sphere) or 45S5 Bioglass^®^/polyethylene composite (Medpor-Plus)	H	HA and alumina implants, being FDA approved since many years, are routinely used in the clinical practice	Naik et al. (2007) and Baino et al. (2014)
			Early uses of 45S5 Bioglass^®^/polyethylene composite spheres in the clinical practice	
Wound healing	45S5 Bioglass^®^/polymer composite meshes	AS	No study involving human patients available	Day et al. (2004) and Rai et al. (2010)
Skin tissue engineering	Fibrous constructs comprising MBG fibers as such or in combination with a polymer	–	No biological study available	Hong et al. (2010) and Jia et al. (2011)
Lung tissue engineering	Sol–gel glass foams or PDLLA/45S5 Bioglass^®^ porous composites	IV	No *in vivo* study available	Tan et al. (2002) and Verrier et al. (2004)
Muscle tissue engineering	Phophate glass fibrous constructs	–	No *in vivo* study available	Ahmed et al. (2004), Shah et al. (2014), and Shah et al. (2015)
Peripheral nerve repair	Bioactive glass fibrous constructs	AS	No study involving human patients available	Vitale-Brovarone et al. (2012a) and Kim et al. (2015)

*H, humans; AS, animal study; IV, *in vitro* tests with cells*.

All the applications of bioactive ceramics take the advantage of bioactivity and minimize mechanical-property requirements, which may be an issue in the case of highly porous implants. Stock porous blocks of various size made of HA, (biphasic) calcium phosphate, and a few bioactive glasses (Table 3) are currently marketed worldwide and clinically implanted in humans for the repair of small bone defects; these implants can be contoured intraoperatively by the surgeon to match the size/shape of the defect. SFF-derived custom-made HA porous scaffolds are produced if a high accuracy on the size or complex shapes are needed, such as implants for orbital floor repair (Levy et al., 1997). Trabecular bone – which can be actually considered a natural bioceramic-based composite – from bone banks is also used as a restorative material (Schlickewei and Schlickewei, 2007).

A special non-osseous application where (porous) bioceramics are widely used is the fabrication of orbital implants for enucleated patients. Porous spherical implants (scaffolds) made of bovine, coralline, and synthetic HA as well as alumina are routinely implanted upon anophthalmic socket surgery as they are biocompatible and allow fibrovascularization within their pore network (Baino et al., 2014). Early human trials with 45S5 Bioglass^®^/polyethylene composite porous orbital implants showed promising results, including an enhanced implant fibrovascularization compared to other available devices (Naik et al., 2007), which can be due to the angiogenic effect of bioactive glass.

In recent years, the use of bioceramic and composite scaffolds – usually comprising bioactive glass as an inorganic phase – has also been proposed for some emerging applications in contact with soft tissues. In this regard, the angiogenic potential of bioactive glasses has opened new perspectives in skin tissue engineering. Day et al. (2004) first showed *in vitro* (using fibroblasts) and *in vivo* (in rats) the ability of 45S5 Bioglass^®^ incorporated into PGA meshes to increase scaffold neovascularization, which would be highly beneficial during the engineering of larger soft tissue constructs. Nano-sized 45S5 Bioglass^®^ particles were also used by Rai et al. (2010) in the fabrication of a novel poly(3-hydroxyoctanoate)-based composite scaffold for wound dressing: the incorporation of bioactive glass nanoparticles accelerated blood clotting time and enhanced the wettability, surface roughness, and overall biocompatibility of the scaffold.

Hong et al. (2010) investigated the use of ultrathin MBG hollow fibers (diameter around 600 nm), fabricated by electrospinning combined with a phase-separation inducing agent [poly(ethylene oxide)], as a multifunctional system for skin tissue engineering (support to the regenerated tissue and release of anti-inflammatory drugs) when organized in the form of 3-D macroporous membranes. MBGs were also mixed with chitosan to produce composite films by freeze–drying for possible use as hemostatic membranes for skin repair (Jia et al., 2011).

Bioactive glass scaffolds have been also proposed for lung tissue engineering applications. In a study by Tan et al. (2002), sol–gel-derived bioactive glass foams with surface modifications to include amine or mercaptan groups and/or coated with laminin were manufactured and placed in culture with murine lung epithelial cells to determine the best conditions to promote cell growth and proliferation. Based on histological examination of the cell cultures, there was full colonization of the foams by the lung cells and it was shown that the laminin-coated, amine-modified foams were most effective in promoting cell growth and attachment.

In another study, Verrier et al. (2004) proposed the use of PDLLA/45S5 Bioglass^®^ porous composites for lung tissue engineering performing *in vitro* biocompatibility assays with a human lung carcinoma A549 cell line. Two hours after cell seeding, a general increase of cell adhesion according to the increased content of Bioglass^®^ (0, 5, and 40 wt.%) in the PDLLA foams was observed, but cell proliferation studies over a period of 4 weeks revealed a better aptitude of A549 cells to proliferate on scaffolds containing only 5 wt.% of glass. These results seem to indicate the possibility of using bioactive glasses in lung tissue engineering approaches, although a lot of future work, including testing with the different cell types found in this complex tissue, is necessary for further advancements.

The results reported by Verrier et al. (2004) demonstrate that the concentration of bioactive glass in tissue engineering polymer-based constructs should be always optimized depending on the considered tissues that we want to regenerate. This dose-dependent effect was also observed in another study by the same research group, in which PLGA/45S5 Bioglass^®^ composite tubular foam scaffolds (porosity about 93 vol.%, size of interconnected macropores in the 50–300 μm range, wall thickness within 1.5–3.0 mm) were fabricated via TIPS (Boccaccini et al., 2005); the authors proposed the use of the produced constructs for the regeneration of tissues requiring a tubular shape scaffold, such as blood vessels and trachea.

The research group led by Prof. Jonathan Knowles also carried out a few studies with phosphate glasses for applications in muscle regeneration. Ahmed et al. (2004) found that CaO–Na_2_O–Fe_2_O_3_–P_2_O_5_ glass fibers allowed attachment, proliferation, and differentiation of conditionally immortal muscle precursor cell line with the formation of myotubes along the axis of the fibers. Shah et al. (2005) found that human masseter-derived cells seeded on a 3-D mesh construct not only attached and proliferated but also migrated along the fibers forming multinucleated myotubes. It was also found that 3-D aligned fiber scaffolds were able to support unidirectional cell alignment and caused an up-regulation of genes encoding for myogenic regulatory factors (Shah et al., 2014), even when the glass fibers were embedded into a collagen gel to form a composite scaffold (Shah et al., 2015). Glass fibers were also found to support and direct axonal regeneration both *in vitro* and *in vivo* (Vitale-Brovarone et al., 2012a; Kim et al., 2015).

Because of their ability to bond to soft tissues and to elicit desirable biological responses, such as angiogenesis, bioactive glasses have been recently proposed in a non-porous form for some other interesting non-osseous applications. A few examples concern the use of bioactive glass particulate for the treatment of gastric ulcers, injectable radioactive glasses for killing cancer cells in liver tumor, glass/polymer composites for cardiac tissue engineering, and glass/polymer tubes for peripheral nerve regeneration. These applications, not restricted to porous scaffolds, have been recently reviewed by some leading scientists in the field (Baino et al., 2016b; Miguez-Pacheco et al., 2015a,b).

## Summary and Outlook

Progress in tissue engineering has led to the development of porous materials designed and manufactured to act as a scaffold for the growth of new tissue in order to restore the natural state and function of diseased parts of the body. Bioceramics have demonstrated to be highly suitable materials for tissue engineering scaffolds and developments in processing methods have provided a mean to control the 3-D architecture of such scaffolds. In spite of remarkable advances, bioceramics have not yet reached their full potential but research is ongoing.

Besides “traditional” use for osseous defect repair, a variety of innovative applications are emerging; for instance, recent studies have interestingly highlighted the suitability of bioactive glasses and glass–ceramics for wound healing applications and soft-tissue engineering (Baino et al., 2016b; Miguez-Pacheco et al., 2015a). For these applications, where softer and more flexible materials are needed, inorganic–organic hybrids could be an even better solution. These materials are interpenetrating networks of inorganic and organic components that interact at a molecular level; they behave as a single phase and, thus, degrade as one material (overcoming the main drawback related to composite biomaterials). Their mechanical properties as well as bioactivity can be tailored by varying the constituents and synthesis/processing parameters (Jones, 2009, 2013).

The use of porous bioceramics as parts of a complex prosthetic devices and not only as a bone-filling material for the restoration of osseous defects but also as a “warm” challenge that has recently arisen. In this regard, a fascinating approach that has been put forward is the use of glass-derived scaffolds as osteointegrative trabecular coatings on ceramic acetabular cup of hip joint prosthesis. These coatings are expected to induce biological fixation of the prosthesis while eliminating the need for invasive screws, cements, or threading to fix implants in place (Vitale-Brovarone et al., 2012b; Baino and Vitale-Brovarone, 2015b; Baino et al., 2015, 2016a).

The development of multifunctional bioceramics that combine the “conventional” properties of 3-D porous bioactive scaffolds and the added value of therapeutic ion release also has great potential. In this regard, bioactive glasses can be doped with various trace elements to provide a smart strategy for the controlled delivery of ions *in situ*, such as Sr, Cu, Zn, Ga, or Co, which may lead to therapeutic effects upon their release into the cellular environment (e.g., promotion of angiogenesis, antibacterial action) (Hoppe et al., 2011; Mourino et al., 2012).

Fabrication of bioceramic components with hierarchical porosity has also recently attracted the interest of biomaterials scientists (Colombo et al., 2010). The use of MBGs either in the form of macro-/mesoporous scaffolds or as coatings can add valuable extra-functionalities to the (base) scaffold. The mesoporous texture and high surface area of these glasses intensify the rate of surface reactions, leading to a faster release of ionic species upon glass dissolution. Therefore, not only the classical bioactivity mechanism is speeded up (fast formation of a surface layer of HA that allows strong bone bonding *in vivo*) but also therapeutic metal ions, previously incorporated within the glass network, can be quickly released upon contact with biological fluids (Wu and Chang, 2014). The solubility rate of MBGs can be tailored by controlling the textural parameters (e.g., mesopore structure and size) and by changing the glass composition so that they dissolve at controlled rates matching those of the tissue growth. A further added value is using MBGs as carriers for the controlled delivery of drug molecules that can be incorporated in the material mesopores (Arcos and Vallet-Regí, 2013), thereby creating a multifunctional tissue engineering implantable device.

New strategies for scaffold fabrication are also emerging both to improve the scaffold performance and to develop ever more sustainable processing routes. For instance, highly porous bioactive glass scaffolds were successfully produced by an innovative method based on preceramic polymers containing micro- and nano-sized fillers (Fiocco et al., 2014). Silica from the decomposition of the silicone resins reacted with the oxides deriving from the fillers, yielding glass–ceramic components after heating at 1000°C. Despite the limited mechanical strength, the obtained samples possessed suitable porous architecture and promising biocompatibility and bioactivity, as testified by preliminary *in vitro* tests. This method has also been very recently applied to fabricate wollastonite/diopside composite foams for bone tissue engineering applications (Fiocco et al., 2015).

If an oriented pore microstructure and high mechanical properties are required, freezing of ceramic slurries can represent a valuable, relatively simple strategy to this aim (Liu et al., 2012).

In summary, new, continuous advances in scaffold processing technologies and novel emerging applications of porous scaffolds in both hard- and soft-tissue engineering bring further honor to the long history of ceramics in medicine. We forecast a bright future for bioceramics, which will indeed provide an ever increasing contribution in improving the quality of life of mankind.

## Author Contributions

FB conceived the study, performed literature search and wrote the paper. GN performed literature search and wrote the paper. CV-B wrote the paper. All authors critically revised the manuscript.

## Conflict of Interest Statement

The authors declare that the research was conducted in the absence of any commercial or financial relationships that could be construed as a potential conflict of interest.
